# Challenges and needs among pediatric oncology providers in caring for pediatric brain tumor patients and survivors: A qualitative study

**DOI:** 10.1016/j.ijchp.2026.100698

**Published:** 2026-06-05

**Authors:** Clara Lefebvre, Géraldine Gazzo, Anne-Sophie Defachelles, Cléa El Hakim, Malika Mezloy-Destracque, Leandra Desjardins, Natacha Entz-Werlé, Sandra Raimbault, William Gehin, Kristopher Lamore

**Affiliations:** a Universite de Lille, CNRS, UMR 9193 - SCALab - Sciences Cognitives et Sciences Affectives, Lille F-59000, France; b Pediatric and Adolescents/Young Adults Oncology Unit, Centre Oscar Lambret, Lille 59020, France; c Department of Pediatric Onco-Hematology, University Hospital, Vandoeuvre-les-Nancy 54500, France; d Département de Pédiatrie, Université de Montréal, CHU Sainte-Justine, Axe Maladies Immunitaires et Cancers, Montréal, Quebec, Canada; e UMR CNRS 7021, Translational, Transveral and Therapeutic Oncology (OnKO-3T) Team, University of Strasbourg, Illkirch, France; f Pediatric Onco-Hematology Unit, University Hospitals of Strasbourg, Strasbourg, France; g Radiotherapy Department, Institut de Cancérologie de Lorraine, Vandoeuvre-lès-Nancy, France

**Keywords:** Pediatric brain tumor survivors, Providers, Challenges, Pediatric oncology, Barriers, Cancer

## Abstract

**Context:**

Children with pediatric brain tumors (PBT) follow a unique clinical course as diverse morbidities may emerge during treatment and may even persist into survivorship. Effectively addressing their needs requires joined efforts from various providers in order to provide optimal support.

**Aims:**

To investigate the challenges and needs of providers working in pediatric oncology with PBT patients and survivors.

**Method:**

Providers with experience working with PBT patients were recruited throughout France to participate in a semi-structured interview. Data were analyzed following a reflexive thematic analysis method.

**Results:**

48 providers were interviewed, with a total of 46 participants included in the final study. Main challenges and needs were identified through 6 themes addressing the strained care environment, the triangular relationship with patients and their family, the complex needs of PBT patients, the emotional burden of care, the long journey of the care pathway, and coordination aspects. Main challenges encompassed poor communication among care teams, as well as patient-provider communication. Repeatedly facing poor prognoses and end-of-life situations were also identified as emotional challenges by providers. Participants also mentioned the necessity to decrease administrative task load, to increase the development of multidisciplinary care, and that training regarding specific PBT care needs is essential for providing optimal care.

**Conclusion:**

Pediatric neuro-oncology represents a unique and emotionally demanding field. Despite persistent communication and coordination challenges, findings highlight the critical importance of dedicated, well-coordinated multidisciplinary teams and long-term follow-up care to meet the complex needs of PBT patients and their families*.*

## Introduction

Worldwide, Primary pediatric brain tumors (PBT) are the most frequent solid tumors, accounting for about 25% of cancer diagnoses among children and adolescents (0 to 18 years old; American Cancer Society, 2023), after leukemia. About 3000 youths are diagnosed each year in Europe and nearly 500 per year in France (Gustave Roussy Cancer Campus, 2025). Survival rates of PBT are increasing in European countries (Allemani et al., 2018) (up to 80% in certain cases, such as low-grade astrocytoma) as well as in the rest of the world (Girardi et al., 2019). However, PBT remain one of the main cancer-related cause of death among children (Girardi et al., 2019) and survivorship is often associated with disease- and treatment-related sequelae impacting quality of life (Rey-Casserly & Diver, 2019).

From diagnosis to treatments to long-term follow-ups, PBT survivors and their family members are at risk of elevated post traumatic stress symptoms (Al-Saadi et al., 2022). Indeed, PBT patients and survivors face increased risks of altered neurodevelopmental processes (e.g. motor dysfunctions, hearing and visual impairments), as well as associated endocrine disorders, psychosocial difficulties, fertility problems and neurocognitive impairments (Nicklin et al., 2022; Sharkey et al., 2020). Cognitive challenges may in turn negatively impact academic or professional achievements (Stavinoha et al., 2021) as well as developing and maintaining friendships (Hocking et al., 2020), or even romantic relationships (Ljungman et al., 2022). These experiences can not only affect self-esteem, but also foster symptoms of anxiety and depression (Cheung et al., 2019; Hörnquist et al., 2014; Sharkey et al., 2020). Difficulties encountered by PBT patients and survivors also include disappointment when attempting to pursue conventional milestones, thus forcing them to reconsider their educational or professional ambitions (Law et al., 2022). Together, the many unique needs and difficulties experienced by PBT survivors require ongoing and comprehensive follow-up by multiple providers (Stavinoha et al., 2018).

Various providers are committed to addressing the multiple short- and long-term sequelae affecting PBT patients and survivors, such as physicians (e.g., pediatric oncologists, radiation oncologists, general practitioners) and nurses, but also speech therapists, teachers, psychologists, neuropsychologists, and social workers. These pediatric oncology providers experience high rates of occupational stress, particularly in two main domains: (1) communication with patients and family; (2) organizational variables such as a hierachical system, workload, shortages and low support (Zarenti et al. 2021). While multidisciplinary teams of providers are recommended to provide optimized and coordinated care to PBT patients (Steven et al., 2019; Fischer et al., 2016), poor communication between and within teams is frequently reported as contributing to a stressful work environment and a barrier to effective communication in pediatric oncology (Odeniyi et al., 2017). Still, most providers in pediatric oncology perceive communication as crucial (Laronne et al., 2022). In fact, trauma-informed communication has been reported by nurses in pediatric oncology as a useful resource to provide developmentally appropriate and family-centered care (Çinar Özbay et al., 2025). Nonetheless, various barriers impeding effective communication exist, including a lack of training and emotional distress experienced by providers (Laronne et al., 2022). Indeed, nurses in pediatric oncology are particularly exposed to moral distress, which has been identified as a predictor for burn-out, poorer communication and decision-making processes in end-of-life situations, as well as less compassionate care provided to patients (Eche et al., 2023). Pediatric oncology providers report positive impacts related to their work on their lives, such as greater ease in developing relationships, psychological empowerment or greater spirituality (Semerçi et al., 2021). However, repeated exposition to traumatic issues can also negatively impacting their lived experience at work and outside of it. In turn, such consequences can lead to persistent thoughts about death, despair, cancer or fear of having a child (Semerçi et al., 2021).

In addition, perceptions of the care provided in pediatric oncology may vary across providers and disagreements may arise. Such conflicts may reflect the need for improvement in functioning of multidisciplinary teams to ensure optimal care is provided to patients and their families (Dalberg et al., 2013).

Furthermore, providers frequently face additional pervasive stressors such as organizational issues, including healthcare and hospital administrative task loads, management, as well as a lack of adapted physical spaces and training resources or frequent rotation among clinicians (Kiwanuka et al., 2019; Sisk et al., 2022). Future conceptual models of care in pediatric neuro-oncology, similar to those developed in pediatric palliative care for instance (Kaye et al., 2017), may advocate for optimized multidisciplinary and embedded care specific to PBT patients. To do so and to identify opportunities to ensure improved care and to better support those who are providing it, we need a better understanding of the complexity of providers’ experiences in pediatric neuro-oncology.

To our knowledge, there is no study addressing the challenges and needs of providers caring for PBT patients and survivors. Hence, the aim of this study is to explore challenges and needs experienced by these providers in their daily practice caring for patients and survivors with a PBT.

## Methods

### Study design

The study followed a qualitative research method, including semi-structured interviews. Qualitative data were reported using the COREQ (Consolidated Criteria for Reporting Qualitative Studies) guidelines (Tong et al., 2007).

### Population

To comprehensively capture the variety of lived experiences from the multidisciplinary backgrounds of providers caring for PBT patients, the study was addressed to providers working in pediatric oncology or in an adolescents and young adults (AYA) department. These included physicians (e.g., pediatricians, surgeons, radiation oncologists, radiologists, psychiatrist, etc.), physical activity instructors, physiotherapists, nurses, psychologists, social workers, etc. To be eligible, providers had to (1) be working with patients under 25 years old, (2) have patients that received a diagnosis of PBT before the age of 18, and (3) understand and speak French fluently. No additional exclusion criteria were pre-specified; however, interviews were excluded from analysis when the participant’s discourse did not focus on pediatric PBT care. Participants were recruited from January 2025 to May 2025 from pediatric oncology departments across France, encompassing comprehensive cancer centers, as well as public hospitals. Using a chain-referral and purposive sampling approach, a broad range of perspectives from providers typically involved in multidisciplinary PBT care across France and from various services of comprehensive cancer centers were captured. As a result, we were able to grasp the perspectives of professionals with diverse backgrounds, in terms of experience and training, who share the same goal of caring for PBT patients (e.g. oncologists, nurses, surgeons, psychologists).

Recruitment was conducted using a mailing list which included eligible providers working in France, and through a LinkedIn post. To reach data thematic saturation regarding participants’ experiences, researchers aimed to recruit 30 to 40 participants (Guest et al., 2020).

Participants received a reminder email one or two days before the interview.

### Procedure and ethical agreement

Each participant received an information letter before the interview. All interviews were confidential, and each participant was attributed a code to preserve anonymity. Interviews were done remotely and occurred only once with each participant. We recommended to participants to be alone in a private room during the interview. Interviews were conducted by a female member of the research team, composed of a PhD student in psychology (CL), a post-doctoral researcher with a PhD in neuroscience (GG), or a master student in psychology (CEH). Interviews ranged from 30 minutes to an hour. Interviews were recorded and later transcribed verbatim using Whisper (AI software). Each interviewer made field notes following every interview and eventually used them as a reminder of the course of the interview to prevent potential interpretation bias of data.

On the day of the interview, individuals were reminded of the study purpose and verbal consent was obtained before proceeding. Researchers and participants did not know each other before the study. Participants were informed about the interviewer employment. Interviews started with the collection of socio-demographic data, including: gender, age, employment, years working with PBT patients, working place (e.g., public or private hospital, comprehensive cancer care center), and unit (e.g., pediatric department and/or AYA unit). An interview guide was developed (see Table 1) by CL according to clinical observations and previous qualitative studies investigating challenges and needs of healthcare providers in oncology (Howard et al., 2018; Lopez et al., 2021). Interviews conducted by investigators (CL, GG or CEH) addressed various domains: perceived positive or negative aspects of the medical care proposed to patients; perceived needs of patients; what providers needed to improve PBT medical care; tools required to address the needs of patients or their own. This study was conducted with the approval of the Ethics Review Committee of the University of Lille (number 2024-831-S133) and in accordance with the General Data Protection Regulation.

**Table 1:** Interview Guide.

**Starting interview with: “Could you please describe your experience regarding the care proposed to your patients with a PBT?”**	
**Main domains explored**	**Follow-up phrases**
Perceived positive or negative aspects of the medical care proposed to patients	*How would you describe/characterize your relationship with patients and their families?* *Could you describe how you and/or your team work to care for your patients? What are the roles of each member of the team?* *Can you tell me about any patients you have treated who have made a particular impression on you, for good or bad reasons, and explain why?* *What is your opinion on the care offered to young patients with brain tumors?* *Do you encounter difficulties in caring for your patients with brain tumors? And their families?*
Perceived needs of patients	*What support needs do you perceive in your patients? And in their families?*
What participants needed to improve medical care	*In your daily work, do you encounter difficulties in supporting children with brain tumors and their parents?* *Since you started working in this field, how do you think the care of children with cancer has changed? What are your thoughts on this?*
Tools participants required to address the needs of patients or their own	*Given what we discussed about XXX, what tools/support/proposals would you have liked to have had in this situation?*

### Data analysis

A reflexive thematic analysis was performed by a PhD student in psychology (CL) and a postdoctoral student in psychology with a PhD in neuroscience (GG), following Braun and Clarke methodology (2021). A data-driven inductive approach was adopted, while acknowledging that the authors’ subjectivity and experiences were inherently intertwined to the analysis process. The diverse background of researchers (psychology and neuroscience) highly contributed to the researchers’ positionality in the data analysis process. One researcher was more familiar with adult psycho-oncology and research methods in psychology (CL), such as semi-structured interviews, and adopted a rather more latently and experiential point of view approach of data analysis (Braun et al., 2022). Combined with the other researcher’s more critical and semantical approach (GG) and rich experiences in health sciences, these two backgrounds constituted one of the core components of the reflexive thematic analysis conducted.

The analysis followed six key phases: (1) familiarization with the data through repeated reading of transcripts, (2) generation of initial codes, (3) searching for potential themes by collating codes, (4) reviewing and refining themes, (5) defining and naming themes, and (6) producing the final report. Codes were drawn from examination of patterns and recurring words emerging in each participant’s discourse, which conveyed a shared meaning. Themes were identified from collating codes conveying occurrences and similarities regarding the participants’ individual but mutual experiences.

One researcher (CL) analyzed all the interviews, while the other (GG) analyzed 40 interviews. Transcribed interviews were not returned to participants.

To reduce potential biases in interpretation and ensure that analytic conclusions emerged from data rather than being imposed by researchers, analyses were first conducted independently. Following this, multiple meetings were organized and assisted by a third senior researcher (KL) familiar with thematic analysis, but exterior to the interviews, who supervised data triangulation in the 3^rd^, 4^th^ and 5^th^ phases of analysis. Disagreements were discussed between the two initial researchers during these meetings. If they were unable to reach a consensus, the third senior researcher was responsible for ruling on the disagreement.

### Member checking

Once all interviews were analyzed and themes were identified, results were shared with participants following the member checking method (Lincoln & Guba, 1985). In addition to data triangulation and field notes from researchers, this procedure was used to support validity through participants’ confirmation of the credibility of the study results (Creswell & Miller, 2000). In the present study, we followed the 5 steps of the synthesized member checking method developed by Birt et al. 2016.

## Results

### Participants

Out of the 887 providers approached for this study, 48 participated in semi-structured interviews. Due to a lack of sufficient neuro-surgeons in pediatrics, neuro-surgeons from adult oncology departments occasionally assist their pediatric counterpart in a selected number of surgeries. This practice eventually led our research team to mistakenly include two participants whose care focused rather on the young adult population with a brain tumor than pediatrics. Consequently, these two interviews were excluded from analysis because their content did not include pediatric care. The final sample is therefore composed of 46 participants.

Detailed participant characteristics are presented in Table 2. Non-participants were providers who did not answer our invitation or who were no longer working in oncology or in pediatrics. Participants predominantly identified as women (39/46; 84.8%), and nurses^1^ (7/46; 15.2%) as well as oncologists (5/46; 10.87%) were the most frequent profession. Mean age of participants was 44.1 years old (SD: 8.89; Range: 28-62). Participants had a mean working experience in pediatric oncology of 14.62 years (SD: 9.25; Range: 1-40).

**Table 2:** Descriptive characteristics.

		**M**	**SD (Range)**
**Age**		44.1	8.89 (28-62)
**Years working with PBT**		14.62	9.25 (1-40)
		**N**	**%**
**Gender**			
	Female	39	84.78
	Male	7	15.21
**Employment**			
	Adapted physical activity instructor	1	2.17
	Advanced practice nurse	2	4.35
	Art therapist	1	2.17
	Clinical psychologist	4	8.70
	Coordinating nurse	4	8.70
	Healthcare nurse manager	2	4.35
	Medical assistant	1	2.17
	Neuropsychologist	2^1^	4.35
	Nurse	2	4.35
	Pathologist	1	2.17
	Pediatric neurosurgeon	3	6.52
	Pediatric nurse	3	6.52
	Pediatric oncologist/hematologist	5	10.87
	Pediatrician	3	6.52
	Pharmacist	1	2.17
	Radiation oncologist	4	8.70
	Radiology technician	2	4.35
	Hospital school teacher	1	2.17
	Social worker	2	4.35
	Socio-aesthetician	1	2.17
	Special education teacher	1	2.17
**Workplace**			
	Comprehensive Cancer Care Center	17	36.96
	Public Hospital	29	63.04
**Department of expertise**			
	Pediatrics	23	50
	Pediatrics and AYA	11	23.91
	AYA	4	8.70
	Radiation therapy	6	13.04
	Other	2	4.35

PBT: Pediatric Brain Tumors

1 One participant was both a clinical psychologist and a neuropsychologist

### Thematic analysis

The following section describes the 6 themes inductively identified from the data collected: (1) A strained care environment; (2) Navigating through the triangular relationship; (3) Complex needs require tailored care; (4) Riding the hill of emotional challenges; (5) Hurrying up in a slow-paced environment; (6) Coordination, both a resource and an obstacle. Throughout the presentation of these six themes, the challenges and needs encountered in caring for PBT patients are presented. Table 3 provides a detailed overview of these themes, including verbatims.

**Table 3:** Verbatims from identified themes.

**(Theme 1) A strained care environment**	
**Subthemes**	**Illustrative quotations**
The administrative framework	*“And that's something I'd really like to see change, but hey, that's the way the administration works, and yeah, sometimes everything takes time, and we don't get any help. And it's frustrating.” (Radiation oncologist, pro 45)*
Shortages	*“The last [psychologist] in place had personal issues and that's why she left. But it's true that, yeah, we have trouble retaining people. But over the last three or four years, it's mainly because management isn't making the necessary efforts to pay its employees decently. There you go, I'll say it plainly, both for psychologists and for the rest of the hospital staff.” (Hematologist-oncologist, pro 7)*
Help from charities	*"Regarding cognitive assessment materials, sometimes we don’t always have the most up-to-date equipment… and we have to buy it again, and the hospital actually won’t cover it. For example, many of the tests I use for assessments are paid for by a charity linked to the department. It’s an association within the department, dedicated to the service, and they’re the ones who pay for my materials." (Psychologist, pro 47)*
**(Theme 2) Navigating through the triangular relationship**	
**Subthemes**	**Illustrative quotations**
Representation of the disease and treatments	*“It is still a very noble organ that represents a great deal in people's minds, which is normal. And so, just the thought of operating on their child's brain is frightening.” (Radiation oncologist, pro 45)*
Care tailored to the family and its specificities pre-existing to the illness	*“Yes, that's right. When illness strikes in social and family situations that are already fragile for various reasons, it makes things even more complicated.” (Social worker, pro 43)*
A privileged relationship	*“As a result, caregivers can work more calmly; social workers, sports coach, nurses, and doctors can work more calmly because a climate of trust has been established. And it's also much more peaceful for the child and the parent. Everyone ends up feeling better about the situation.” (Socio-esthetician, pro 37)*
Emotional response from parents	*“In any case, I try not to get upset when parents raise their voice or become a little aggressive because I tell myself that it's not personal, that it's really the situation they're going through that's pushing them to their limits, and that's normal, and that there's no point in confronting them.” (Radiation oncologist, pro 45)*
Place of parents in the treatment process	*“We meet parents who know almost more than I do about how things work, with chemotherapy, PACs, stem cells, and so on. So they are almost more knowledgeable than I am.” (Neurosurgeon, pro 4)*
Trauma from treatment	*“But coming, well coming back to the hospital all the time, I think that for some children, I think that the fact that they could potentially be treated elsewhere gives them a new perspective, and I would say that, yes, my difficulties will be more pronounced there.” (Psychologist & Neuropsychologist, pro 47)*
**(Theme 3) Complex needs require tailored care**	
**Subthemes**	**Illustrative quotations**
Special considerations in pediatrics: patients are children first and foremost	*“Because he's still a child first and foremost. Yes, he's sick, but he's still a child” (Adapted Physical Activity Teacher, pro 35)*
Comparisons / A specific expertise?	*“And then I think my colleagues may also have fewer patients who die or who are in these situations. I'm thinking in particular of my colleagues who treat leukemia and lymphoma, which are indeed diseases that respond well to treatment.” (Pediatric oncologist, pro 11)*
Multidisciplinary expert teams on PBT	*“It [the treatment of pediatric brain tumors] is really, um, well, for me, there are two terms that describe it: it's really, um... it's really multidisciplinary because the repercussions of the disease will impact all areas of the patient's life.” (Pediatric nurse, pro 27)*
Several long-term sequelae	*“But we do know that these patients are more likely to develop cardiovascular disease, kidney failure, secondary cancer, and so on, so they need special monitoring.” (Nurse/Health executive, pro 13)*
Psychosocial care	*“You can mention the word “tumor” and they immediately think of death, perhaps because a loved one is... their grandmother died, I don't know, they see hospitals. In short, it's true that we have a psychologist who is always there, and I think she does an extraordinary job.” (Neurosurgeon, pro 4)*
Specificities of radiotherapy	*“There have been various cases, it's a bit difficult to talk about them, because they're often quite traumatic situations where we've... There was one case in particular, well, several cases where we forced the child. That is to say, we gathered a bunch of adults around them, sat them down, forced them to put the restraint casts on, and then tied them up after all that, and they screamed throughout the entire session. We end up having to do this, which is traumatic for them, but it's also traumatic for the team who does it.” (Radiology technician, pro 39)*
**(Theme 4) Riding the hill of emotional challenges**	
**Subthemes**	**Illustrative quotations**
Emotionally challenging care	*“I have stories where I struggle... where I struggle to hold back, except for the tears. But, um... I... I empathize deeply with patients.” (Neurosurgeon, pro 4)*
End-of-life, relapse and palliative care	*“Sometimes you see them come back two or three times and you don't see them getting better, but rather getting worse. Often you know that the outcome of their illness is... well, neurological tumors in children are rarely... well, it rarely ends well.” (Radiation therapy technician, pro 38)*
Knowing treatments' costs	*"But it's true that when I'm at the hospital and I come out of a follow-up consultation, and I see all the consequences of our treatment, I think to myself, um... well, I wonder if this is really the right thing to do." (Radiation oncologist, pro 45)*
Delivering bad news	*“We still make diagnosis announcements... and... we... say the word, the word tumor, and of course you can imagine what the reaction is. There's crying, it's immediate as soon as they understand. And so these are very delicate moments, um... where we sometimes have to try not to cry ourselves, because there are situations that are a little...” (Neurosurgeon, pro 4)*
Healthcare provider-patient/parent communication	*“Because things aren't... aren't understood at all and... Um... Pons, DIPG for example, where um... They come out of the consultation, it's a horrible tumor. I don't know if you're familiar with it, but I guess you must have looked into it a bit. Um... Where the pediatrician announces the... the illness at the same time as announcing palliative care. It's, um... Well, sometimes they don't hear the part about palliative care.” (Nurse/Health executive, pro 13)*
Communication resources	*“And typically, these are situations where my colleague, the doctor, likes me to be there, partly because it's difficult to find the right words, it's difficult to know when the parents are ready to hear the news.” (Psychologist, pro 42)*
Difficulty in taking advantage of support programs	*“Afterwards, it's not because there's a psychologist that people would go and see her, I think. For example, we hold post-death meetings... and quite often, not many people come because even if these are situations that raised questions at the time, well, with hindsight, people move on.” (Pediatrician, pro 5)*
**(Theme 5) Hurrying up in a slow-paced environment**	
**Subthemes**	**Illustrative quotations**
Long-term progression of the disease	*“Sometimes it's complicated because these are... these are patients who, even though they're in care, let's say they're in palliative care, it can last for a very long time, and then you really see physical deterioration, and that, I think, is one of the hardest things, actually, physical deterioration that lasts and lasts and lasts because, well, they... ...well, despite everything, they hang on, and that can be hard, yeah. In any case, it's been hard for me, I think it can be hard for the nursing staff.” (Advanced practice nurse, pro 41)*
Rapid onset of the disease	*“There is a difference compared to before, when the news came as a shock and death occurred very quickly. Now, the news is still just as shocking, but the deterioration is much more gradual, which changes the way we care for these children and their families.” (Pediatric nurse, pro 17)*
Supporting patients takes time	*“First of all, because I said so, there are multiple issues to work on, more so in this case than in others. These issues need to be worked on over the long term, longer than others, longer than patients who have had other types of cancer. Um... and sometimes these patients have... concentration problems, memory problems, and so on, which means that we need to spend more time working with them, and that's not easy.” (Coordinating nurse, pro 27)*
Emergence and evolution of side effects throughout the course of life	*“Indeed, in certain situations, these difficulties will worsen over time, unlike in certain cases where they tend to stabilize or even improve.” (Pediatric nurse, pro 27)*
**(6) Coordination, both a resource and an obstacle**	
**Subthemes**	**Illustrative quotations**
Multidisciplinary communication	*" Sometimes there are communication issues, because there are several institutions involved in the care of the same patient... that's it. We always make sure to include everyone in the email loop or the phone call loop. But since there are so many people involved in the care of these children, sometimes communication issues can arise.” (Medical assistant, pro 33)*
Centralized network management	*“So first of all, that means that these are patients who sometimes live far away, um... and that's a special circumstance because it makes it more difficult to treat and monitor these patients, and often, um, when treatment is finished, these are patients we can lose, and even though, um, cancer treatment is coming to an end, it doesn't mean that these children's care journey is over, because, well, depending on the type of tumor, depending on what it may have caused in terms of post-surgery disability, or post-cancer disability, there is this follow-up that is difficult to ensure because the patients are spread out over such a large area that we sometimes lose track of them.” (Psychologist, pro 34)*
Home-based medical care	*“In brain tumors, we see this [patients receiving essentially home-based medical care] a lot: patients who only come in for consultations and therefore miss out on a very rich support system. Even if it’s just nursing care, where nurses are able to build relationships with children and families through their daily care, providing them with plenty of time for conversation, human contact, games, and many other things.” (Pediatric oncologist, pro 29)*
Coordination and consistency of care	*“I would say that coordination, anything that could give a sense of consistency, will be important. In fact, the child will be there, and we will involve many, many providers around them. Sometimes it will continue over the long term, sometimes it will stop. I would say that if we could have a kind of consistency, a stronger regularity.” (Psychologist, pro 34)*

#### *Theme 1 – A strained care environment*

This theme combines the multiple administrative burdens and shortages, particularly staffing shortages, providers endure while caring for their patients. Many participants reported that management was not aligned with the realities of caring for PBT patients, leading to frustration. Participants mentioned frustration as a common state while navigating situations where shortages, such as a lack of office or spaces, are a daily reminder of their inability to provide the ideal care they wished to offer to their patients and their families:

“It frustrates teams because we're not doing a good job; we're doing what we can with the resources we have, and that's frustrating for the teams.” (*Nurse, pro 8*)

Moreover, participants reported being slowed down in their practice because of compelling and growing administrative workload across time, exacerbating feelings of frustration and helplessness (see Table 3):

“It's very annoying to have barriers that are absurd. Dealing with absurdity is quite difficult. Sometimes.” (*Radiation oncologist, pro 10*)

Also, participants described precarious contractual work situations characterized by low wages while having demanding work hours, and also short-term contracts or part-time employment (see Table 3). Staffing and material shortages, as well as space constraints, were a core component of the daily practice of providers, and considerably exacerbated the state of frustration of participants. The gaps between ideal care and actual care involved difficult access to spaces dedicated to PBT patients (within the hospital but also outside, such as rehabilitation centers), understaffing of qualified providers, lack of time and missing materials (e.g., leaflets to communicate about the disease, neuropsychological or psychological assessment material). Regarding the latter, although some participants related that pediatric oncology is well furnished, others reported a gap between what they needed in order to offer tailored and ideal care and what they had. Some participants also mentioned creating their own tools, while others received financial help from charity to pay for furniture or testing materials (see Table 3):

“We’re told there’s no budget. It’s the public hospital, there’s no budget. I need a cabinet to store my files? Well, it’s the association that buys it for me. That’s how it is!” (*Psychologist, pro 47*)

#### *Theme 2 – Navigating through the triangular relationship*

This theme captures how participants managed the relationship with patients and their families, considering each family enters the service with preexisting family dynamics which may impact the care provided. According to participants, patients and families often had a vivid representation of PBT as a rare and deadly disease affecting a vital organ (see Table 3):

“Just saying “tumor” is one thing, but saying “brain tumor” is something else entirely.” (*Nurse, pro 27*)

Participants also reported providing tailored care that considers the socio-economic and cultural context of the family, thus reinforcing the necessity to be in a constant adaptative mode (see Table 3). According to participants, the disease forces parents to mourn the child they knew before the brain tumor and ultimately disturbes the family balance. At the same time, participants perceived the development of a privileged relationship between themselves, the parents and the patient. This triangular relationship in pediatrics was a core component of participants’ daily work. They described how they were constantly trying to find the right proximity while building a trust-based relationship and how the latter could be a helping resource or a burden if trust was undermined (see Table 3). In this context, while knowing the gravity of the situation and having in mind the prognosis of the PBT, participants were repeatedly engaging in emotional regulation with parents, sometimes facing uncertainty, other times hope, but also at times, violence and aggressivity:

“So you have to be realistic while trying not to make them lose hope, while trying to be clear and not lie to the child, but also not put ideas into their head because you don't know what's going on in their mind.” (*Neurosurgeon, pro 4*)

Concurrently, participants described how much parents became actors in the care delivered to their child, especially during home-based hospitalization. Participants offered education and reassurance to parents in regards to providing adapted care (see Table 3).

In the end, participants reported how traumatizing treatments could be for patients and their family who sometimes did not want to come back to the hospital. Consequently, they had to find alternatives to provide follow-up care (see Table 3).

#### *Theme 3 – Complex needs require tailored care*

This theme reflects how much the care provided to PBT patients is adjusted to their specificities, whether during or after treatment. Participants highlighted specificities of working in a pediatric department which included, above all, considering patients as children who are eager to maintain their school life, to make friends and to reach other typical developmental milestones:

“Also, how can we ensure that a child continues to be a child when they are confined, while they are sick? Finally, how can we not forget that, before being sick, they are children who have the same needs as any other child?” (*Social worker, pro 43*)

Oftentimes, the care provided to PBT patients was compared to the care of other pediatric patients or to adult patients in oncology (see Table 3). No participants’ practice was solely dedicated to PBT patients. Through comparison of their experiences across diverse patient populations, they highlighted the expertise required in taking care of PBT patients and their family (see Table 3). Some expressed their wish to benefit from more training in pediatrics, on how to take care of children and on how to approach the relationship with parents. Others expressed the need for general education about PBT and treatments administered in pediatrics.

Moreover, participants emphasized how much treatments can be disabling and the challenge it is for providers to address the multiplicity of long-term sequelae patients may experience. For this reason, several participants pointed out the necessity to develop multidisciplinary expert teams dedicated to PBT patients and survivors (see Table 3):

“It's hard to find good professionals to take care of our children because they don't fit into the mold.” (*Neuropsychologist, pro 44*)

In this context, participants also mentioned that psychosocial care is essential to address the variety of difficulties encountered by patients through lifelong treatments (see Table 3). Psychosocial needs were regularly pointed out by participants, whether their own or those of patients and their families. Participants, and especially radiotherapy providers, reported the difficulty they could encounter to immobilize patients, eventually triggering trauma-like feelings in providers following tumultuous situations (see Table 3).

#### *Theme 4 – Riding the hill of emotional challenges*

Participants described how caring for PBT patients and their families created considerable emotional burden. Some expressed that working with these patients was hard because they repeatedly faced end-of-life or relapse situations where they felt helpless due to lack of treatment options left to offer (see Table 3):

“The stage where we are all still somewhat helpless is when we get to palliative care.” (*Neurosurgeon, pro 9*)

Still, participants reported feeling divided at times when they proposed curative treatments which they knew would ultimately cause considerable morbidity for patients (see Table 3):

“So I think to myself, okay, we cured them, but at what cost in the end?” (*Radiation oncologist, pro 45*)

Communication between participants, patients and families was identified as a common difficulty, notably because it often involved delivering bad news, such as diagnoses or poor prognoses (see Table 3). Lack of communication triggered stress for participants, for instance when they lacked time to effectively exchange with their patients or because families were perceived as being not in a good position to hear and understand the information. In addition, participants mentioned that families were increasingly confronting them with (false) information about the disease from the internet (see Table 3). Participants found it demanding to explain to desperate (and sometimes aggressive) parents that this online content was not applicable to their child’s case.

To ease the burden of delivering bad news, some participants reported using strategies such as attending consultation in pairs (see Table 3). Although emotional challenges was a very commonly reported difficulty, several participants declared being unable or not willing to use available help. For instance, many mentioned not wanting to attend sessions with a psychologist, whether these sessions were offered within their institution or not. Others declared that they tried but did not find the help they wished for, or that they would rather discuss with colleagues (see Table 3):

“There remains... the preconceived notion that psychologists are for weak people, or rather, “it's not for me, I don't need it, I'm so strong, I don't need a psychologist.”” (*Adapted Physical Activity Teacher, pro 35*)

#### *Theme 5 – Hurrying up in a slow-paced environment*

This theme conveys the pressure experienced by participants when the diagnosis is delivered, which contrasts with the clinical course of many PBTs that can extend over months or years, requiring ongoing monitoring and support. In addition, it was not rare for participants to take care of patients for whom no curative treatments were available. Still the evolution of the tumor was carefully monitored over the years, as is done for patients with chronic illnesses (see Table 3):

“So, in brain tumors, we have certain tumors that... well, in certain oncological pathways, brain tumors are a chronic disease trajectory.” (*Pediatrician, pro 25*)

Participants described that it takes time to address the complex care balance between the rapid onset of the disease and its sometimes slow evolution with long-term treatments (see Table 3). They reported that one particularity of PBT is that the side effects resulting from treatments could arise even years after the tumor was treated, and therefore need to be continuously addressed (see Table 3):

“So it's true that it's, it's, these are really patients that we may never see during their course of treatment, and who will contact us again long after.” (*Pediatric nurse, pro 27*)

#### *Theme 6 – Coordination, both a resource and an obstacle*

Almost all participants identified coordination as essential to the care provided to PBT patients and their families. Reasons for coordination issues were the stress experienced when diagnoses were provided, and poor communication among providers within multidisciplinary teams or between providers from different comprehensive cancer care centers (see Table 3):

“It's between the team, which is mainly nurses and paramedics, and the doctor's team. You can sense that sometimes there is a lack of communication.” (*Neuropsychologist, pro 48*)

Also, coordination was perceived by participants as geographically complex, as some treatments, such as radiation therapy, are provided only at some centralized centers across France, often requiring families to travel long distances (see Table 3):

“And there are indeed patients that we will lose [sight of]. So yeah, I would say that the most challenging aspect of these conditions is not being able to provide follow-up care from start to finish.” (*Psychologist, pro 34*)

Coordinated care was not always smoothly provided by participants, especially for patients who received home-based medical care. Even though participants were satisfied that patients had the option to receive medical services at their homes, they described not having many chances to interact with them which consequently, rendered the establishment of a solid and trusting relationship more difficult (see Table 3). In this context, participants mentioned the struggle to provide families a feeling of continuity, coordination and global reassurance (see Table 3):

“Since I've been here, there have been changes in the teams, but I'm still here, so that's also part of it, you know, I'm a constant figure for them, so there's that reassuring aspect to it.” (*Psychologist, pro 40*)

## Discussion

The present study aimed to explore the experience of providers working with PBT patients, and to identify their challenges and needs. As therapeutic options keep advancing in pediatric neuro-oncology, leading to an increase in survival, the care practices of providers must also keep evolving in parallel to the multiple and evolving needs of PBT patients and survivors. Main challenges experienced by our participants included poor communication, poor coordination in a complex care need situation, and facing poor prognoses and end-of-life situations, eventually triggering emotional difficulties. Main needs included a reduced administrative workload, multidisciplinary teams and training dedicated to PBT patients, and better coordination pathways.

### Challenges

Participants frequently reported that poor communication within teams or with other centers involved in the clinical course of patients was an obstacle. Such observations echoe the results from the systematic review of Sisk et al. (2021) on multilevel barriers to communication in pediatric oncology. In the “Team barriers” theme, authors reported obstacles to communication between oncology team and other specialties, as well as within the oncology team in which unclear roles and authority could impede fluid communication. Participants from our study reported similar issues. Indeed, because PBT and their treatments cause multiple short- and long-term sequelae, a wide range of providers work simultaneously together. For instance, with the rapid onset of the disease, some were not informed in time about an incoming patient, which triggered a feeling of irritation. Team communication is an important challenge in pediatric oncology, and more research should address this issue. Still, studies often focus on patient-provider communication rather than issues at the team level (Sisk et al., 2022). Although interventions focusing on increasing communication skills remain rare and heterogeneous in pediatric oncology, they have been identified as beneficial by participating providers and are associated with favorable outcomes, such as: better therapeutic alliance and improved alignment of care between medical objectives and patients and family choices (Kaye et al. 2020). Communication skills training in pediatric neuro-oncology, especially in emotionally challenging situations, such as end-of-life or progression of the disease, could constitute a helpful resource for providers.

Among our participants, the introduction of dedicated coordinating providers seemed to alleviate some communication challenges. PBT patients require specific multidisciplinary expert teams to address complex symptoms and lifetime evolving late effects. While some providers are centralized at a given place, such as radiation oncologists, others may be more geographically dispersed, such as support care like neuropsychologists, speech therapists or psychologists. Centralized care enables providers to offer optimal comprehensive pediatric neuro-oncology care. Still, in a qualitative study by Luckett et al. (2024) on the experience of providers working with PBT patients, participants identified the unequal access to healthcare for patients living in remote areas as a complex and common obstacle. In fact, it impaired long-term follow-ups which are critical to PBT survivorship care.

Considering the specificities of treatments and remission, PBT share similarities with other patients with a chronic illness condition. Whether it is during treatments or survivorship, to some extent PBT patients and survivors appear to meet criteria of “Children with Medical Complexity” (CMC) as categorized by Kingsnorth et al. (2015): (1) they are considered a fragile population with a severe life-threatening disease; (2) their condition may be chronic since it last at least 6 months or more; (3) PBT care involves multiple providers and it is delivered at several locations; (4) PBT patients are users of high instensity care. In this perspective, adopting an integrated complex care model for CMC could improve the coordination and continuity of care. Current care of PBT patients and survivors are often provided according to a comanagement-centered model of care, where providers deliver care-coordinated services at comprehensive care centers (Pordes et al., 2018). Still, PBT care also conforms to other models of complex care for CMC. Current and previous work, such as the report on pediatric complex care coordination by Rosenbaum (2008), are paving the way for further integrated models. A PBT care pathway adjusted following a complex care management model could further assist providers.

Whether it was delivering bad news or providing support, our participants also faced a major challenge when provided care involved emotional issues. A study from Sisk et al. (2018) reported that physicians tended to exchange medical information while being more evasive on discussing emotions, even sometimes deferring emotional support to nurses. Participants from our study also identified patient-provider communication as a substantial challenge, as they reported caring for PBT patients implied frequently delivering bad news and being more often emotionally triggered with end-of-life situations and poor prognoses than for other pediatric malignancies. Indeed, PBT experience and care provided can be traumatic both for patients, family and providers (Çinar Özbay et al., 2025). For providers in pediatric oncology, such end-of-life situations may trigger feelings of despair, pessimism or make them question the purpose of life (Semerçi et al., 2021).

### Needs

Likely not specific to caring for PBT patients and survivors, we identified that increasing administrative workload and precarious work conditions constituted a significant stressor for participants. This observation echoes the work on moral distress, first defined in nursing by Jameton (1993) as knowing the “right thing to do, but institutional constraints make it nearly impossible to pursue the right course of action” and eventually inducing discomfort or suffering. Preventing moral distress among providers is a critical issue, as it has been associated with a desire to leave their position (Mathews et al., 2023). Preventing moral distress in pediatric neuro-oncology requires not only further studies on this topic, but also advocacy efforts to improve working conditions. For instance, in their survey assessing burnout and career satisfaction in neuro-oncology, Yust-Katz et al. (2020) identified nurses, assistants and support care providers as well as basic scientists, before physicians, as being the most at risk for professional burnout. While increased job satisfaction tended to reduce this risk, high emotional exposition and low professional achievements were the most frequently reported predictors of professional burnout. Supplementary survey-based studies in pediatric neuro-oncology would provide a state-of-the-art overview on the subject and stand as data-based evidence in favor of improved working conditions. In addition, further guidance regarding core competencies of hospital managers should be provided, such as evidence-informed decision-making and knowledge of healthcare environment and organization, as identified by Kakemam et al. (2020) in their Management Competency Assessment Program.

Participants also insisted on the relevance of a multidisciplinary team of experts for PBT patients and survivors to address their various late effects. Indeed, PBT are unique diseases requiring lifelong monitoring by multiple care providers with various backgrounds to help optimize quality of life. To some extent, follow-up care is more complex than surveilling tumor recurrence and must be delivered by a robust multidisciplinary team (Fischer et al., 2016; Treadgold et al., 2019). Consequently, specialized team structuration for PBT emerged in comprehensive cancer care hospitals, such as at the Oscar Lambret Center or Gustave Roussy hospitals in France. Still, such multidisciplinary teams exist predominantly within large comprehensive cancer centers, which may lead to health inequalities. Several participants also mentioned they wished for more training on pediatric care and/or on neuro-oncology. This recurring sub-theme seems to echo the desire for more standardized guidelines in pediatric neuro-oncology designed specifically to PBT patients needs and the family-centered care they receive, as formulated by healthcare providers in Australia caring for PBT patients (Luckett et al., 2024). When Lucket et al. (2024) investigated the barriers and facilitators in pediatric neuro-oncology, participants emphasized the frequent adaptation of adult neuro-oncology care to pediatrics. Pediatric and adult neuro-oncology are not merely extensions of one another and require knowledge regarding specific diagnoses and treatment protocols which may be distinct between settings. Therefore, an additional challenge pediatric neuro-oncology team members might face is the need to acquire additional knowledge specific to pediatric neuro-oncology in order to adapt treatments. For instance, one radiation oncologist among our participants emphasized the need for pediatric oncology treatments specifically designed for children rather than adapted from adult oncology treatments: *“So, the list for Santa... We want protocols that apply to both adults and children. We want it so that when we test a new drug... for chemo, we also test radiation therapy in combination with it.”* (Radiation Oncologist, pro 10). Moreover, Lindsay et al. (2023) led a survey-based study on the need to develop a pediatric neuro-oncology training and accreditation in the United States of America, with 75% of the physicians surveyed supporting the implementation of a certification process. Future international collaboration in pediatric neuro-oncology could support sharing the experience of clinical realities and further promote the harmonization of best practices in multidisciplinary PBT care.

### Clinical implications

Considering the variety of sequelae (e.g. cognitive, physical, psychosocial), the evolving lifetime late effects of PBT, and according to our participants’ experiences, we formulated three main recommendations for providers working in pediatric neuro-oncology, that may already exist in some hospitals:

(1) As the necessity of trained multidisciplinary teams of experts in pediatric neuro-oncology has been reported, we recommend formalizing dedicated times for multidisciplinary meetings, involving not only physicans but all providers such as nurses and supportive care providers, from diagnosis to long-term follow-up. We noted some geographical disparities through our interviews which were reported in theme 6 (“Coordination, both a resource and an obstacle”): such teams and meetings may exist in large comprehensive cancer care centers, providers caring for PBT patients and survivors from others hospitals would greatly benefit from this practice.
(2) Increasing the number of nurse coordinators and providing training in interprofessional communication could represent a first step to address coordination of complex care needs. Furthermore, expanding the use of telehealth practice could further support providing care to patients and survivors living in remote areas or reluctant to come back to the hospital after acute treatments, but still in need of long-term follow-up. However, this alternative to in-person follow-up consultations did not seem to fit the expectations of all PBT survivors and their caregivers in the experience of Cacciotti et al. (2023) at the Dana-Farber/Boston Children’s Hospital. In-person consultations were preferred by some survivors and their caregivers as they enabled developing a better personal connection with providers. Hence, the use of telehealth multidisciplinary services for follow-up consultations needs refinement and PBT survivors should be free to choose between in person and remote consultations.
(3) As reported and emphasized by our participants in our theme 4 (“Riding the hill of emotional challenges”), caring for PBT patients exposes providers to highly emotional situations. Therefore, institutions should actively prioritize the mental well-being of pediatric neuro-oncology providers by developing structured, sustainable support mechanisms—such as regular supervision, peer-support programs, or access to specialized psychological services—aimed at preventing emotional exhaustion and supporting providers facing repeated exposure to distressful situations.

## Limitations

To our knowledge, the present study is the only one to date to investigate the experience of providers taking care of PBT patients and their family in France. Although this study aimed to advance knowledge in the limited domain of pediatric neuro-oncology provider experiences, we note that findings are context-specific and may not be transferable to other healthcare settings or countries. We recommend that readers carefully identify dimensions of similarities in terms of times, samples and settings to ensure good transferability to other contexts (Trochim, 2006). Additionally, this work involves limitations such as a self-selection bias from participants. It is more than likely that those who voluntarily agreed to participate were providers already sensitive to the challenges and needs encountered in taking care of PBT patients. Further studies could use longer periods of recruitment to reach eligible participants and recruit participants unfamiliar with the subject. Furthermore, even though we intended to recruit all types of providers, more participants were medical providers (e.g., pediatricians, oncologists, radiation oncologists, nurses) than those providing supportive care (e.g., speech therapists, organizational therapists, neuropsychologists, art therapists). Although we reached data saturation, due to our large sample size, and tried to counterbalance this effect using purposive sampling, it may also constitute a bias to recruitment. In addition, as thematic analysis is inherently reflexive, the researchers’ backgrounds and experiences may have shaped the analysis. To address this, we engaged in reflexive practices, including maintaining field notes and holding regular discussions with a third researcher to critically reflect on coding and theme development. Different researchers might have identified alternative nuances or emphasized different aspects of the data.

## Conclusion

Our study investigated the challenges and needs experienced by providers working with PBT patients, survivors, and families. Multiple late effects and poor prognoses characterized this malignancy, catalyzing communication, coordination, and emotional challenges for providers, as well as highlighting the need for additional training in pediatric neuro-oncology and for multidisciplinary teams of experts of PBT.

## Ethical considerations

The Ethics Review Committee at University of Lille approved our interviews (approval: 2024-831-S133) on Januray 17th, 2025.

## Funding statement

This study is part of the Pediacriex EN-HOPE SMART4CBT consortium, directed by Natacha Entz-Werlé, funded by the 10.13039/501100006364French National Cancer Institute (grant INCa-Cancer_18693); Clara Lefebvre PhD is funded by the 10.13039/501100004099Ligue contre le cancer (grant_18542); Kristopher Lamore working time is funded by the 10.13039/501100006364French National Cancer Institute (Institut National du Cancer, grant INCA/16136) in collaboration with the University of Lille, the SCALab laboratory, the ONCOLille Institute, and the Centre Oscar Lambret that support the research chair opsyrii "innovations in psycho-oncology and intervention research".

## Data availability

The datasets generated and analyzed during the current study are not publicly available. Respondents shared sensitive information about patients. They were assured raw data would remain confidential and not be shared to preserve their anonymity while ensuring that they could speak freely during interview. Still, some data can be available from the corresponding author on request.

## Declaration of competing interest

The authors declare that they have no known competing financial interests or personal relationships that could have appeared to influence the work reported in this paper.

## Footnotes

1 We include here pediatric nurses (n=3), nurses (n=2) and advanced practice nurses (n=2)
